# Different soil respiration responses to litter manipulation in three subtropical successional forests

**DOI:** 10.1038/srep18166

**Published:** 2015-12-11

**Authors:** Tianfeng Han, Wenjuan Huang, Juxiu Liu, Guoyi Zhou, Yin Xiao

**Affiliations:** 1 Key Laboratory of Vegetation Restoration and Management of Degraded Ecosystems, South China Botanical Garden, Chinese Academy of Sciences, Guangzhou, 510650, China

## Abstract

Aboveground litter inputs have been greatly altered by human disturbances and climate change, which have important effects on soil respiration. However, the knowledge of how soil respiration responds to altered litter inputs is limited in tropical and subtropical forests. We conducted an aboveground litterfall manipulation experiment in three successional forests in the subtropics to examine the soil respiration responses to different litter inputs from January 2010 to July 2012. The soil respiration decreased by 35% in the litter exclusion treatments and increased by 77% in the doubled litter additions across all three forests. The reduction in soil respiration induced by the litter exclusion was greatest in the early successional forest, which may be related to a decrease in the soil moisture and shifts in the microbial community. The increase in soil respiration produced by the doubled litter addition was largest in the mature forest, which was most probably due to its relatively high quantity and quality of litterfall. Our results suggest that the effect of reduced litter inputs on the soil respiration lessened with forest succession but that the doubled litter inputs resulted in a stronger priming effect in the mature forest than in the other two forests.

Carbon dioxide (CO_2_) emission from soils through soil respiration is the largest terrestrial carbon (C) flux contributing to atmospheric CO_2_^1^. Soil respiration is very sensitive to environmental changes because it can be affected by a variety of factors, such as soil temperature, soil moisture^2^, microbial community, soil surface litter and vegetation types^3^,^4^. A small change in soil respiration can have profound impacts on the global C balance and consequent feedbacks to climate change^5^. Therefore, soil respiration in response to environmental changes must be understood.

Aboveground litterfall regulates energy flow and nutrient cycling in forest ecosystems^6^. Human activity has increasingly altered litter inputs to soils. For instance, extensive deforestation and cultivation are likely to decrease litter inputs^7^, whereas elevated CO_2_ often increases litterfall^8^. Changes in aboveground litter inputs can potentially affect soil respiration through the direct decomposition of litterfall and indirect effects on biological processes in the underlying soil^9^,^10^,^11^. Litter removal may reduce decomposition of soil organic C due to decreasing the easily decomposable substrate for microbes^12^. Increased litter inputs may increase soil CO_2_ emissions in temperate forests due to the priming effects^13^,^14^. Conversely, Fekete *et al.*^4^ have reported that soil respiration did not increase with doubled litter inputs in a Central European deciduous forest because decomposition was hindered by low soil moisture. Most studies on the response of soil respiration to litter inputs, however, are limited to temperate forests. Tropical and subtropical forests store and cycle a large amount of C^15^,^16^, and their responses to litterfall inputs may be different from those of temperate forests^17^. Thus, improving our understanding of the vulnerability of tropical and subtropical forest ecosystems to environmental changes is imperative to better predict future atmospheric CO_2_ concentration.

Recent research on the lowland tropical forests in Panama has shown an enhancement of soil C release by litter addition, and this effect continued for several years^18^. Leff *et al.*^17^ have found that soil CO_2_ efflux was affected by two years of litter manipulation treatments in tropical forests, with a relatively greater decline in litter removal than an increase in litter addition. Given the diversity of tropical and subtropical forest ecosystems^19^, however, the response of soil respiration to litter inputs may be different among forest types due to the different abiotic (soil temperature and moisture) and biotic (the quality and quantity of the aboveground litter inputs) factors^20^,^21^,^22^. Previous studies have reported that the effects of litter manipulation on soil microclimate (soil temperature and moisture) vary with forest types^23^, which would potentially influence the soil respiration responses. Moreover, changes in the plant community composition may shift the accessible C and nutrient stoichiometry in their litter inputs^24^, which consequently influences soil respiration. Through laboratory incubations, Whitaker *et al.*^25^ have found that the soil respiration increased to a greater degree with inputs of microbially accessible C compounds than with complex, recalcitrant ones. However, previous studies on soil respiration in response to environmental changes have often focused on a single forest type. Few studies have compared the soil respiration responses to environmental changes among different forest types, especially those with different successional stages.

In this study, we chose a regional forest community, subtropical monsoon evergreen broadleaf forest (BF), and its two successional forests of mixed pine and broadleaf forest (MF) and pine forest (PF) at the Dinghushan Biosphere Reserve. These distinct forest types allowed us to examine the effects of the forest types on soil respiration in response to the aboveground litterfall changes subjected to the same regional climate. Our recent work has shown that the annual litterfall in the three forests has been altered over the last 30 years, with a decrease in the BF, no change in the MF and an increase in the PF^26^. Under the changing litterfall inputs, the accumulation rate of soil organic C during the same period was greater in the BF than in the PF and MF^27^. The progressive C accumulation in soils with forest succession was related to the faster litter decomposition and the lower loss of litter mass through respiration in the BF than in the other two forests^28^. These findings highlight the differences in the litterfall and/or its chemical components controlling the soil C dynamics among the three forests. Therefore, the objective of this study was to investigate the effects of litter manipulation (litter removal and litter addition) on the soil respiration, the soil temperature and moisture, the soil microbial community and the soil C in the three forests. We hypothesized that (1) litter removal would decrease soil respiration, whereas litter addition would increase it and (2) the effects of the litter inputs on soil respiration would vary with forest succession.

## Results

### Soil temperature and moisture

The wet season (from April to September) showed higher soil temperature and soil moisture than the dry season (from October to March of the following year). In the control, the mean soil temperature in both the dry and wet seasons was significantly higher in the PF and MF than in the BF. In the dry season, the mean soil moisture in the control was as follows: PF (17.1%) < MF (20.8%) < BF (26.4%); the moisture differences among the forest types were significant. In the wet season, the mean soil moisture was significantly lower in the PF (20.9%) than in the MF (27.8%) and BF (28.7%). Litter manipulation failed to significantly affect the soil temperature and moisture in the three forests (Table 1). However, litter exclusion in the PF tended to decrease the annual mean soil moisture (*P* = 0.077), especially in the dry season (*P* = 0.093).

### Soil respiration

**Figure 1:**
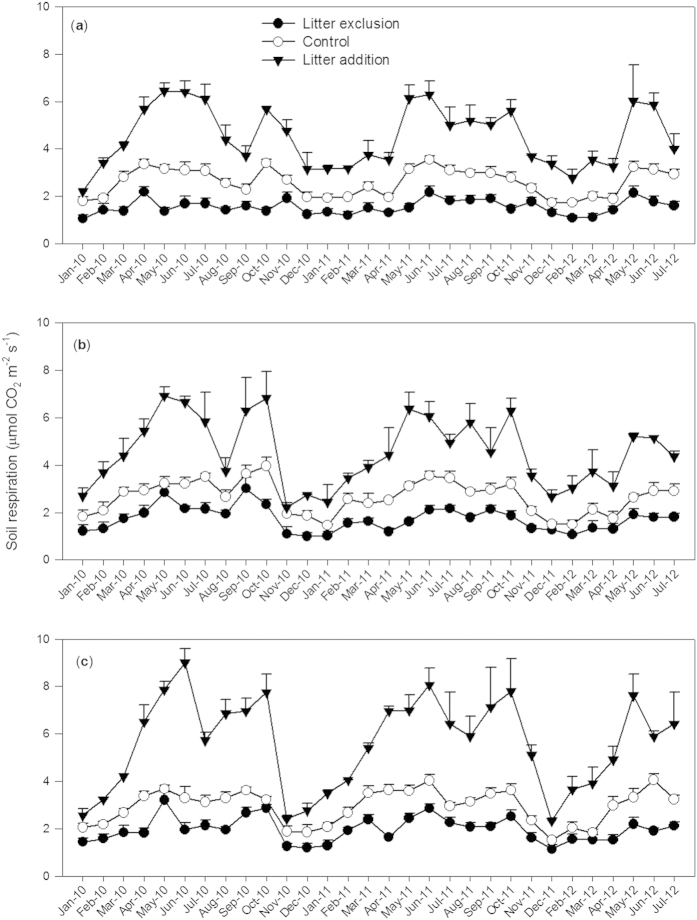
Dynamics of soil respiration in the three forests exposed to different litter manipulation treatments from January 2010 to July 2012. (**a**) Pine forest (PF), (**b**) Mixed pine and broadleaf forest (MF), and (**c**) Monsoon evergreen broadleaf forest (BF). Data are Means ± SE.

The three forests displayed a seasonal pattern of soil respiration, with significantly lower values in the dry season than in the wet season (Fig. 1 and Table 1). The soil respiration in the control was significantly higher in the BF than in the MF and PF. The soil respiration was significantly decreased by litter exclusion and significantly increased by litter addition in all three forests (Fig. 1 and Table 1). The contribution to the total soil respiration from recent aboveground litter, estimated from the difference in the soil respiration between the control and litter exclusion plots, did not show any significant differences among the three forests in either the dry or wet season (Table 1). However, compared to the control, the degree of the changes induced by litter manipulation varied with the forest types. In the dry season, litter exclusion decreased the soil respiration in the BF to a smaller extent than in the PF. The recent aboveground litter contribution rate was 39% in the PF, 34% in the MF and 32% in the BF throughout the experimental period, and the value was significantly lower in the BF than in the PF. The increase in the soil respiration induced by litter addition was significantly greater in the BF than in the MF and PF in the wet season and across the entire experimental period. On average, litter addition increased the soil respiration by 72% in the PF, 71% in the MF and 87% in the BF.

### Relationships between soil respiration and soil temperature and moisture

Significant exponential relationships were shown between the soil respiration and soil temperature in all of the treatments in the three forests (Table 2). The mean temperature sensitivity (*Q*_10_) value in the control was 1.31 in the PF, 1.46 in the MF and 1.46 in the BF. The litter manipulation did not significantly affect the *Q*_10_ in the MF and PF, but the litter addition significantly increased the *Q*_10_ in the BF compared to the control. The soil respiration exhibited significantly positive linear relationships with soil moisture in both the control and the litter addition in all three forests. In the case of litter exclusion, the soil respiration was strongly related to the soil moisture in the BF but not in the MF or PF (Table 2).

### Soil microbial community

**Figure 2:**
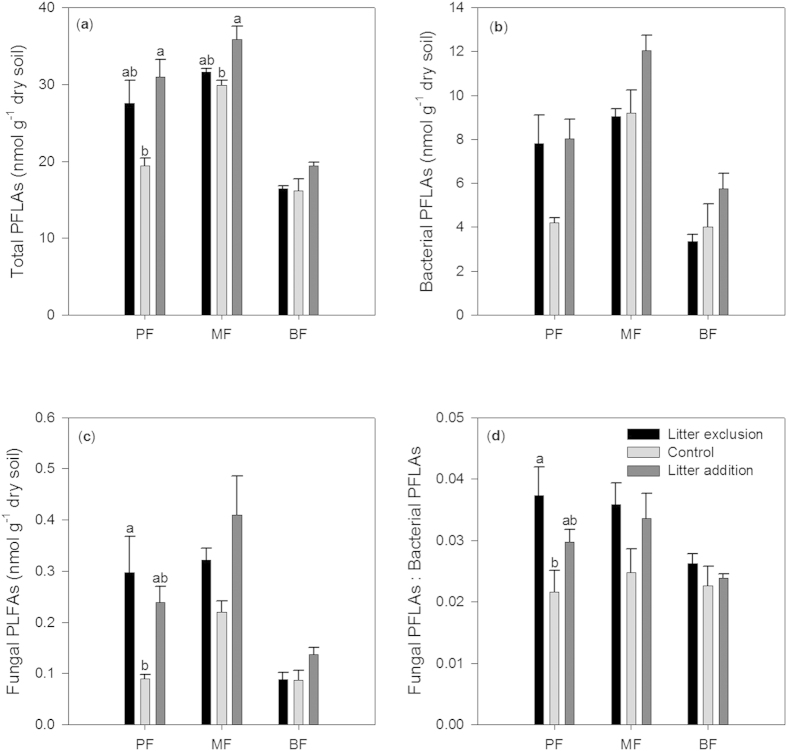
Soil microbial PLFAs in the three forests under litter manipulation treatments in June 2012. (**a**) Total PLFAs, (**b**) Bacterial PLFAs, (**c**) Fungal PLFAs, and (**d**) The ratio of fungal PLFAs to bacterial PLFAs. Data are Means ± SE. Different lowercase letters indicate significant differences among treatments in each forest (*P*  <  0.05).

Litter exclusion significantly increased the fungal phospholipid fatty acids (PLFAs) and the fungi to bacteria ratio in the PF, whereas litter addition significantly increased the total PLFAs in the PF and MF (Fig. 2). However, in the BF, the soil microbial community was not altered by either litter exclusion or litter addition.

### Soil organic carbon

Litter manipulation did not significantly influence the soil organic C in either the PF or MF. In the BF, the soil organic C was significantly higher in the case of litter addition than in litter exclusion (Table 3).

## Discussion

### Effects of litter exclusion on soil respiration

As hypothesized, litter exclusion decreased the soil respiration, which was consistent with results from other studies in tropical and subtropical forests^12^,^17^,^29^. The reduction (35% on average) in the soil respiration due to litter exclusion was similar to the value (28%) measured by a static chamber method at an adjacent site^23^. However, the decrease in soil respiration was much lower than the values reported by Li *et al.*^21^ in wet tropical forests. The differences may be attributed to the more prolonged period of litter removal before the measurement of the soil CO_2_ efflux in the study by Li *et al.*^21^ (6 years) than in the study reported here (2 months).

The withdrawal of fresh substrate is believed to be responsible for reducing the soil respiration in the three forests. However, the extent of the decrease induced by litter exclusion was the greatest in the PF, followed by the MF and BF. Soil respiration includes root respiration and microbial respiration from the decomposition of soil organic matter derived from aboveground and belowground litter^30^. The fraction of the net primary production allocated belowground has been shown to increase with progressive successional stages^3^, corresponding to increased phosphorus (P) limitation in the mature forest^31^. Thus, our findings imply that the influence of belowground inputs on soil C dynamics would be greater in later successional forests.

Litter exclusion usually decreases soil moisture^32^. In the PF, we found that the soil moisture tended to decrease with litter exclusion, especially in the dry season. Lower soil moisture reduces nutrient transport and the metabolism of decomposing microbes^33^,^34^, thus resulting in a greater decrease in soil respiration. However, the soil temperature and moisture were insignificantly affected by litter exclusion in both the MF and BF, thus making little contribution to changes in the soil respiration. In addition, we found that the soil microbial community in the PF was changed by litter exclusion, resulting in a relatively greater ratio of fungi to bacteria. Compared with the MF and BF, the PF was considered to be more limited by nitrogen (N)^35^. The increased dominance of fungi in an N-limited system can result in a lower soil respiration rate because fungi generally have greater C assimilation efficiencies than bacteria (i.e., fungi store more C than they metabolize)^36^. Thus, the changed microbial community under litter exclusion could also partly explain the greater decrease in soil respiration in the PF than in the other two forests.

### Effects of litter addition on soil respiration

Conversely to litter exclusion, litter addition significantly stimulated soil respiration in all three forests. This finding is in accordance with the results of other studies, which have shown that additional litter provided easily decomposable substrate to microbes and thus increased microbial respiration^10^,^13^,^14^. The increases in soil respiration induced by litter addition (77% on average) outpaced the decrease by litter exclusion (35%), indicating that the increased losses of CO_2_ from soils cannot be attributed to litter C addition alone. Some studies suggested that litter addition increased the amount of labile C inputs and also led to priming effects^29^, whereby additional soil organic C mineralization was stimulated by the addition of fresh organic matter^37^. Our results provided strong evidence that priming effects did occur under the litter addition in the three subtropical forests.

We found that the litter addition had a stronger effect on soil respiration in the BF than in the MF and PF. It is unlikely that the greater response of the soil respiration in the BF was caused by the soil temperature, soil moisture or soil microbial biomass and community, because these factors were not significantly affected by the litter addition. Instead, the quantity and quality of the litterfall may be responsible for the different magnitudes of the effects. The mean annual litterfall was relatively greater in the BF (849 g m^−2^) and MF (861 g m^−2^) than in the PF (356 g m^−2^)^38^. We observed that the litter quality was highest in the BF but lowest in the PF (Table 4). The litter addition significantly accelerated the litter decomposition in the BF but not in the MF or PF, further resulting in greater nutrient (especially P) release from the litter in the BF^39^. Therefore, more C and nutrient inputs from the litter addition in the BF could lead to greater soil microbial activity. Moreover, the priming effects can be driven by nutrient limitation in tropical forest soils when soil microbes have sufficient energy^40^. The P limitation in the BF^31^ could possibly result in stronger priming effects for organic P mineralization, especially in the wet season with high biological activity but low soil available P^41^. This effect is also clearly reflected by an experiment of P fertilization that showed that P addition significantly stimulated soil respiration in the BF^42^.

### Implication for soil organic C storage

The soil organic C did not significantly change after approximately two and a half years of litter manipulation, although the soil respiration was altered. A decrease in the soil C with aboveground litter removal has frequently been observed in the Detritus Input and Removal Treatments (DIRT) experiment, which has lasted for decades^43^,^44^,^45^. The unaltered soil organic C in our study may be attributed to the relatively short duration of treatments. The increase in soil respiration induced by the litter addition resulted in considerable CO_2_ emission from the soils in the three forests due to the priming effects. However, the priming effects may be short lived^46^, and thus the accumulation of the litter inputs might eventually increase the soil C if the double-litter treatment were to continue for decades^47^. Further research is needed to assess the contributions of the aboveground litter inputs to the soil organic C and the sustainability of the priming effects.

The litter inputs failed to affect the environmental factors (e.g., soil temperature and moisture), but they caused functional changes in the soil respiration, defined as changes in the soil respiration model parameters in relation to the soil temperature and moisture^48^. Specifically, the litter exclusion led to no significant relationship between the soil respiration and soil moisture in the MF and PF, which reflected that the soil microbial activity was more limited by energy. The soil temperature sensitivity (*Q*_10_) was significantly increased by litter addition in the BF, which was likely due to the increased substrate availability from the litter inputs^49^. Therefore, our results have potential importance for terrestrial ecosystem models to predict soil C dynamics in response to climate change.

## Methods

### Site description

The study was conducted at the Dinghushan Biosphere Reserve (DBR) (23°09′21″N – 23°11′30″ N, 112°30′39″E – 112°33′41″ E) in Southern China. The DBR, covering an area of 1155 ha, was acknowledged as the first National Natural Reserve and was established in 1956 to protect subtropical natural forests in southern China. This region is characterized by a typical subtropical monsoon humid climate. The mean annual temperature is 22.3 °C. The highest monthly mean temperature is 28.0 °C in July, and the lowest is 12.6 °C in January. The average annual precipitation is 1680 mm, of which 80% occurs in the period from April to September, creating distinct wet and dry seasons. The annual average relative humidity is 78%. The bedrocks of the DBR are sandstone and shale belonging to the Devonian Period. The soil is mainly lateritic, classified in the Ultisol group and Udult subgroup according to the USDA soil classification system^50^. The soil pH ranged from 4.0 to 4.9.

Three forest types, PF, MF and BF with respective ages of approximately 50, 100 and more than 400 years, are typically observed at the DBR. They represent a sequence of successional stages from pioneer to climax vegetation communities. The biomass was 40.6 Mg C ha^−1^ in the PF, 116.2 Mg C ha^−1^ in the MF and 147.8 Mg C ha^−1^ in the BF^23^, and the basal area was 24.8 m^2^ ha^−1^ in the PF, 25.4 m^2^ ha^−1^ in the MF and 38.2 m^2^ ha^−1^ in the BF^42^. *Pinus massoniana* Lamb. was initially planted after clear-cutting approximately 50 years ago in the PF (early stage), which was gradually invaded by *Schima superba* Gardn et Champ. The dominant species in the PF, however, is *Pinus massoniana* Lamb., accounting for approximately 90% of the community biomass^31^. The MF (middle stage) has developed naturally from the PF. The dominant species in the MF are *Castanopsis chinensis* (Spreng.) Hance, *Schima superba* and *Pinus massoniana*, composing approximately 50.9%, 25.0% and 18.8% of the community biomass, respectively^31^. The BF (late stage) has not been disturbed for over 400 years. The dominant species in the BF are *Castanopsis chinensis, Schima superba, Cryptocarya concinna* Hance, *Cryptocarya chinensis* (Hance) Hemsl., *Aporosa yunnanensis* (Pax & K. Hoffm.) F. P. Metcalf, *Acmena acuminatissima* (Blume) Merr. et Perry and *Gironniera subaequalis* Planch. These species account for more than 60% of the community biomass^31^. The mean annual litterfall production during the period of 1982–2001 was 356, 861, 849 g m^−2^ for the PF, MF and BF, respectively^38^. The chemical properties of the litterfall in the three forests measured in 2012 are shown in Table 4.

### Experimental design

The litter manipulation treatments included the control (CK), the aboveground litter exclusion (NL) and doubled aboveground litter addition (DL). In each forest, the CK was established in July 2009, and the plots of NL and DL were established in October 2009. There were 9 replicates for the CK, 6 for the NL and 3 for the DL, with a total of 18 plots in each forest. Each plot was 0.6 by 0.6 m to be consistent with a root trenching experiment^51^ for the future comparison of different inputs on the soil C dynamics. The distance between the plots was at least 3 m to avoid overlapping the effects of the different treatments. The CK plots received the normal input of aboveground litter. In the NL plots, litter was excluded with 1 mm mesh screens placed approximately 1 m aboveground. The collected litter from the NL plots was removed monthly and redistributed on the DL plots.

### Soil CO_2_ flux measurement

Soil respiration was measured using the Li-8100 Automated Soil CO_2_ Flux System (Li-Cor Inc., Lincoln, NE, USA). To measure the soil CO_2_ flux, one polyvinyl chloride (PVC) soil collar (20 cm in diameter and 10 cm high) was permanently inserted into the mineral soil in the centre of each plot to avoid edge effects. The insertion depth was 2–3 cm to minimize disturbance to the shallow fine roots. All of the living plants inside the soil collars were removed by hand at least one day prior to the measurements to exclude plant respiration from the aboveground elements. From January 2010 to July 2012, soil CO_2_ effluxes were measured every two weeks in the wet season and once a month in the dry season, depending on the weather conditions. Following a consistent measurement protocol, the soil CO_2_ flux between 9:00 am and 12:00 am on a clear day represents a one-day average value according to the diurnal soil CO_2_ flux variation measured at an adjacent site^24^. The soil temperature (°C) and soil moisture (volumetric water content, %) at 10 cm depth were simultaneously monitored adjacent to each soil collar using a digital thermometer and an MPKit^23^, respectively.

### Soil sampling

The soils were sampled in June 2012. For each plot, three soil cores (2.5 cm inner diameter) were collected from 0–10 cm of soil depth and at least 10 cm away from the PVC soil collar or the plot edges. The collected soils were combined to gain one composite sample per plot. After removing stones and coarse roots, the samples were sieved with 2 mm mesh and divided into two parts. One part of each sample was air dried to analyse the soil organic C, and the other was stored at −20 °C prior to analysing the soil microbial community.

### Soil organic carbon and soil microbial community

The soil organic C was determined by titration with a Fe^2 + ^solution after dichromate oxidation^52^. The soil microbial community was determined using phospholipid fatty acid (PLFA) analysis as described by Bossio and Scow^53^. For each sample, different PLFAs were considered to be representative of different groups of soil micro-organisms. The abundance of the individual fatty acids was determined as relative nmol per g of dry soil and standard nomenclature was used^54^. Bacteria were considered to be represented by 15 PLFAs (14:0, i14:0, 15:0, i15:0, a15:0, 15:0 3OH, i16:0, 16:1 2OH, 16:1ω7c, 17:0, i17:0, a17:0, cy17:0, 18:1ω7c, and cy19:0ω8c), and fungi were considered to be represented by the PLFAs 18:2ω6, 9^55^. The sum of all of these PLFAs plus 16:1ω5c, 10Me 16:0, 10Me 17:0 and 10Me 18:0 was used as an estimation of the total microbial biomass. In addition, the ratio of fungal to bacterial fatty acids was also included in the data analysis. This ratio has often been used as an indicator of the change in the soil microbial community structure^55^.

### Data analysis

Repeated measures analysis of variances (RM ANOVA) was used to test the differences in the soil respiration, soil temperature and soil moisture among the seasons, forests and litter manipulation treatments. The Tukey multiple comparison test was conducted if significant differences among the treatments were found. We assumed that the soil C stores were at a steady state over the short term and that the litterfall C input is equivalent to the respiration by decomposing the recent and previously deposited aboveground litter^11^,^13^. The soil respiration from the recent aboveground litter was calculated as follows: the recent aboveground litter respiration = control plots – litter exclusion plots^11^,^12^. The recent aboveground litter contribution rate was calculated by dividing the recent aboveground litter respiration by the soil respiration in the control. One-way ANOVA with the Tukey multiple comparison test was used to test the differences in the soil respiration changes induced by litter manipulation treatments among the forests. The differences in the soil organic C and the soil microbial community among the treatments in each forest were analysed by one-way ANOVA with the Tukey multiple comparison test.

The nonlinear and linear regression models were used to formulate the relationships of soil respiration with soil temperature and moisture^23^, respectively: 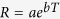 and 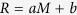, where *R* is soil respiration (μmol CO_2_ m^−2^ s^−1^), *T* is soil temperature (°C), *M* is soil moisture (%), and *a* and *b* are parameters fitted to the regression models. The temperature sensitivity 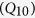 was calculated over the range of temperatures at 10–30 °C. All of the analyses were conducted with R software (R Foundation for Statistical Computing, Vienna, Austria)^56^.

## Additional Information

**How to cite this article**: Han, T. *et al.* Different soil respiration responses to litter manipulation in three subtropical successional forests. *Sci. Rep.* **5**, 18166; doi: 10.1038/srep18166 (2015).

## Figures and Tables

**Table 1:** Mean values of soil temperature, soil moisture and soil respiration in the three forests exposed to different litter manipulation treatments from January 2010 to July 2012.

**Season**	**Forest**	**Soil temperature (°C)**			**Soil moisture (%)**			**Soil respiration (μmol CO**_**2**_ **m**^−**2**^ **s**^−**1**^)			**Recent aboveground litter respiration**
		**NL**	**CK**	**DL**	**DL**	**CK**	**DL**	**NL**	**CK**	**DL**	**(μmol CO**_**2**_ **m**^−**2**^ **s**^−**1**^)
Dry	PF	18.7 ± 0.5	18.2 ± 0.3	18.6 ± 0.6	14.7 ± 0.8	17.1 ± 0.8	17.2 ± 1.0	1.38 ± 0.05 c	2.17 ± 0.06 b	3.74 ± 0.17 a	0.88 ± 0.12
	MF	17.3 ± 0.4	17.5 ± 0.3	17.1 ± 0.6	18.5 ± 1.1	20.8 ± 0.9	19.9 ± 1.5	1.38 ± 0.07 c	2.23 ± 0.09 b	3.69 ± 0.24 a	0.82 ± 0.10
	BF	16.4 ± 0.4	16.2 ± 0.3	16.3 ± 0.5	26.1 ± 1.1	26.4 ± 0.9	25.2 ± 1.4	1.74 ± 0.08 c	2.44 ± 0.08 b	4.19 ± 0.29 a	0.66 ± 0.07
Wet	PF	25.0 ± 0.3	24.8 ± 0.2	24.7 ± 0.4	19.5 ± 0.7	20.9 ± 0.6	19.5 ± 1.0	1.73 ± 0.06 c	2.97 ± 0.06 b	5.34 ± 0.18 a	1.19 ± 0.09
	MF	24.2 ± 0.3	24.0 ± 0.2	24.0 ± 0.4	26.6 ± 0.8	27.8 ± 0.6	27.3 ± 1.1	2.05 ± 0.08 c	3.04 ± 0.07 b	5.56 ± 0.21 a	1.00 ± 0.09
	BF	23.1 ± 0.3	23.1 ± 0.3	22.8 ± 0.5	27.1 ± 0.7	28.7 ± 0.6	27.6 ± 1.0	2.23 ± 0.07 c	3.44 ± 0.07 b	6.83 ± 0.20 a	1.25 ± 0.11

Data are Means ± SE. Different lowercase letters indicate significant differences among the treatments in each forest (*P* < 0.05). NL: litter exclusion; CK: control; and DL: litter addition.

**Table 2:** Model parameters describing the relationships between soil respiration and soil temperature and moisture in the three forests exposed to different litter manipulation treatments from January 2010 to July 2012.

**Model**	**Treatment**	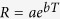	***P***	***b***	***P***	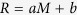		***P***	***b***	***P***
**Forest**		***a***				***Q***_**10**_	***a***			
PF	NL	1.001 ± 0.2311	<0.001	0.0198 ± 0.0097	0.042	1.22	0.0108 ± 0.0071	0.129	1.3784 ± 0.1333	<0.001
	CK	1.4227 ± 0.173	<0.001	0.0272 ± 0.0051	<0.001	1.31	0.0274 ± 0.0074	<0.001	2.0905 ± 0.1506	<0.001
	DL	2.2412 ± 0.2419	<0.001	0.0331 ± 0.0045	<0.001	1.39	0.086 ± 0.0234	<0.001	3.1264 ± 0.4648	<0.001
MF	NL	0.7077 ± 0.1741	<0.001	0.0414 ± 0.0105	<0.001	1.51	0.0154 ± 0.008	0.055	1.3669 ± 0.1957	<0.001
	CK	1.1968 ± 0.1584	<0.001	0.0377 ± 0.0057	<0.001	1.46	0.0309 ± 0.0078	<0.001	1.9457 ± 0.2043	<0.001
	DL	1.8004 ± 0.2137	<0.001	0.045 ± 0.0051	<0.001	1.57	0.0905 ± 0.0242	<0.001	2.7096 ± 0.6275	<0.001
BF	NL	0.9866 ± 0.2048	<0.001	0.035 ± 0.0094 b	<0.001	1.42	0.0248 ± 0.0086	0.005	1.3607 ± 0.2406	<0.001
	CK	1.4119 ± 0.163	<0.001	0.0375 ± 0.0052 b	<0.001	1.46	0.0318 ± 0.0102	0.002	2.1996 ± 0.2941	<0.001
	DL	1.8681 ± 0.1967	<0.001	0.0541 ± 0.0047 a	<0.001	1.72	0.0951 ± 0.0346	0.008	2.988 ± 0.9452	0.002

Data are Means ± SE. NL: litter exclusion; CK: control; and DL: litter addition.

**Table 3:** Soil organic carbon (%) in the three forests under litter manipulation treatments in June 2012.

	**Litter exclusion**	**Control**	**Litter addition**
PF	1.76 ± 0.30	1.62 ± 0.16	2.09 ± 0.23
MF	3.84 ± 0.42	4.82 ± 0.62	5.70 ± 0.44
BF	2.31 ± 0.13 b	2.53 ± 0.22 ab	3.48 ± 0.29 a

Data are Means ± SE. Different lowercase letters indicate significant differences among treatments in each forest (*P* < 0.05).

**Table 4:** Chemical properties of the litterfall in the three forests.

**Forest**	**C (mg g**^−**1**^)	**N (mg g**^−**1**^)	**P (mg g**^−**1**^)	**C:N**	**C:P**
PF	523 ± 8	9.10 ± 0.79 b	0.17 ± 0.01 b	58 ± 4 a	52 ± 2 a
MF	483 ± 11	12.25 ± 0.34 b	0.36 ± 0.03 b	40 ± 2 b	37 ± 4 ab
BF	482 ± 22	17.02 ± 0.92 a	0.60 ± 0.06 a	30 ± 3 b	32 ± 3 b

Data are Means ± SE. Different lowercase letters indicate significant differences among forests (*P* < 0.05). C: carbon; N: nitrogen; and P: phosphorus.
